# Redefining Sudden Cardiac Death Risk Stratification in Dilated Cardiomyopathy: An Integrative Approach

**DOI:** 10.31083/RCM49889

**Published:** 2026-07-14

**Authors:** Aikaterini-Eleftheria Karanikola, Ioannis Doundoulakis, Christos-Konstantinos Antoniou, Panagiotis Alexandrou, Christos Vosikas, Georgios Leventopoulos, Athanasios Kordalis, Dimitrios Tsiachris, Konstantinos Tsioufis, Konstantinos Gatzoulis

**Affiliations:** ^1^First Cardiology Department, National and Kapodistrian University of Athens, Hippokration General Hospital, 11527 Athens, Greece; ^2^Department of Cardiology, University Hospital of Patras, 26504 Patras, Greece

**Keywords:** dilated cardiomyopathy, sudden cardiac death, risk assessment, ventricular tachycardia, genetic testing, magnetic resonance imaging

## Abstract

Dilated cardiomyopathy (DCM) is one of the most common cardiomyopathy phenotypes. Moreover, sudden cardiac death (SCD) remains a leading cause of mortality in patients with DCM, predominantly driven by malignant ventricular arrhythmias (VAs). Current guideline-recommended risk stratification relies heavily on left ventricular ejection fraction (LVEF) to guide implantable cardioverter-defibrillator (ICD) therapy; however, attention has recently shifted toward a multiparametric strategy that integrates clinical characteristics with electrocardiographic markers, biomarkers, cardiac magnetic resonance (CMR) findings, genetic testing, and electrophysiological studies. This review summarizes current evidence on established and emerging tools for SCD risk stratification in DCM, highlights the limitations of single-parameter approaches, and discusses future directions toward more personalized, dynamic risk assessment to optimize SCD outcomes.

## 1. Introduction

Dilated cardiomyopathy (DCM) is defined as the presence of left ventricular (LV) dilatation and systolic dysfunction with left ventricular ejection fraction (LVEF) <50% that cannot be attributed to abnormal loading conditions or coronary artery disease [1]. DCM is a heterogeneous disorder with both genetic and acquired etiologies, including infectious, peripartum, autoimmune, toxic, and drug-related causes [1]. It represents one of the most common cardiomyopathy phenotypes, with a prevalence estimated at approximately 1:250–500 in the adult population and a substantially lower prevalence in children, comparable to that of hypertrophic cardiomyopathy [2].

DCM is a major contributor to sudden cardiac death (SCD), with an annual incidence of approximately 2–4%, predominantly driven by malignant ventricular arrhythmias (VAs) [3]. Landmark randomized controlled trials (RCTs), including DEFINITE and DANISH, have evaluated the role of prophylactic implantable cardioverter-defibrillator (ICD) therapy in patients with non-ischemic cardiomyopathy (NICM) and symptomatic heart failure. These studies enrolled patients with LVEF ≤35% and additional risk markers such as elevated N-terminal pro–brain natriuretic peptide (NT-proBNP), premature ventricular complexes (PVCs), or non-sustained ventricular tachycardia (NSVT), and demonstrated a significant reduction in SCD without a corresponding reduction in all-cause mortality [4,5]. Until recently, guidelines primarily relied on reduced LVEF and New York Heart Association (NYHA) class for SCD risk stratification and decision-making on ICD therapy, yet this approach has managed to identify only a small proportion of patients who truly benefit from primary SCD prevention [6]. Importantly, SCD events also occur in patients with only mildly or moderately reduced, or even preserved LVEF [7]. The aforementioned findings have further highlighted the limitations of LVEF-based risk stratification and underscore the need for more accurate predictive tools [8].

In recent years, attention has shifted toward multimodal approaches that integrate clinical variables with advanced imaging, genetic profiling, electrophysiological testing, and novel biomarkers to improve individualized risk stratification [9]. This comprehensive review summarizes the evolving landscape of SCD risk stratification in DCM and discusses emerging tools and future directions aimed at optimizing personalized preventive strategies.

## 2. Pathophysiology of SCD in DCM

In DCM patients, SCD is essentially driven by malignant VAs, including sustained monomorphic ventricular tachycardia (VT), polymorphic VT, and ventricular fibrillation (VF). Myocardial fibrosis—arising from chronic inflammation, microvascular ischemia, genetic factors, or immune-metabolic abnormalities—is a key substrate predisposing DCM patients to SCD; the replacement of myocytes with collagen and other fibrotic tissue creates heterogeneous, slow-conducting zones within the ventricular myocardium, facilitating reentry circuits and promoting VAs [10,11]. In addition, impaired calcium homeostasis due to specific gene mutations, including Phospholamban (PLN) and histidine-rich calcium-binding protein (HRC), may lead to changes in the action potential duration (APD) and increase arrhythmogenicity [12,13].

An important distinguishing factor of VAs in DCM compared to ischemic cardiomyopathy (ICM) is the frequent epicardial origin of arrhythmogenic foci. These VAs commonly arise from the lateral subepicardial and anteroseptal intramural regions, reflecting the presence of diffuse and patchy fibrosis [14]. High-density electroanatomical mapping studies have provided valuable insights into the complex arrhythmogenic substrate in this population. In a cohort of 66 patients, a significantly higher unipolar-to-bipolar scar ratio was observed in NICM compared with ICM, a finding thought to indicate an imbalanced arrhythmogenic substrate with predominant involvement of the intramural and epicardial layers [15].

The role of epicardial adipose tissue (EAT) and intramyocardial fat is increasingly recognized in the pathophysiology of VAs in patients with NICM. In a cohort of 98 patients with ICM and NICM, CT-derived volumes of both EAT -particularly in a distance of <2.5 mm from the epicardium- and intramyocardial fat were strongly associated with VAs in the NICM group. Notably, a key distinguishing feature between NICM and ICM was the diffuse distribution of intramyocardial fat observed in NICM, in contrast to the more localized, infarct-related pattern seen in ICM [16]. Higher EAT volumes were also observed in carriers of the PLN p.(Arg14del) variant who developed major VAs in a follow-up of 70 ± 35 months [17]. EAT has been proposed to promote arrhythmogenicity by prolonging action potential duration and increasing afterdepolarizations and resting membrane potential, mediated through alterations in calcium metabolism and increased production of reactive oxygen species. In addition, adipocyte infiltration into the myocardium is thought to contribute to structural remodeling and fibrosis [18].

Finally, the autonomic nervous system plays a central role in promoting VAs and SCD in this population, with altered heart rate variability reflecting heightened sympathetic activity and reduced parasympathetic tone [19,20]. Early nuclear imaging studies demonstrated both global and regional impairment of cardiac sympathetic innervation in patients with idiopathic DCM [21]. In this setting, myocyte electrical remodeling appears to act synergistically with heterogeneous sympathetic innervation to facilitate ventricular arrhythmogenesis [22].

## 3. Current Risk Stratification Approaches

### 3.1 Guideline-Based Risk Stratification

Current recommendations for SCD prevention in DCM are largely based on LVEF and symptomatic status. According to the 2022 European Society of Cardiology (ESC) guidelines for ventricular arrhythmias and the prevention of SCD, as well as the 2023 ESC cardiomyopathy guidelines, ICD therapy as primary prevention of SCD is recommended in patients with DCM, LVEF ≤35% and symptomatic heart failure despite optimal medical therapy [6,23]. Importantly, these guidelines also acknowledge the role of additional risk modifiers, including late gadolinium enhancement (LGE) on cardiac magnetic resonance (CMR), high-risk genetic variants and unexplained syncope, which may justify ICD consideration even in patients with moderately reduced or preserved LVEF [6,23].

Similarly, American guidelines support ICD implantation for primary prevention in patients with non-ischemic cardiomyopathy and LVEF ≤35% despite guideline-directed medical therapy, while also emphasizing individualized risk assessment [24]. Notably, both European and US guidelines recognize the limitations of an LVEF-based approach and highlight the need for multiparametric models that incorporate imaging, genetic, and clinical markers to improve patient selection.

In this context, contemporary SCD risk stratification in DCM relies on a multifactorial evaluation that integrates clinical, imaging, laboratory, electrophysiological, and genetic data, with each modality providing complementary insights into the arrhythmic substrate and individual patient risk (Table 1).

**Table 1.** **Risk stratification tools in DCM**.

Clinical parameters	ECG/NIRFs	Genetics	CMR	Biomarkers	EPS/PVS
NYHA class	PVC burden*	LMNA*	LGE presence*	NT-proBNP*	Inducible VT/VF*
LVEF*	NSVT*	FLNC*	LGE extent*	sST2	Sinus node disease
Unexplained syncope*	TWA*	DSP*	LGE patterns*	hs-troponin*	Atrioventricular node disease
Family history of cardiomyopathy/SCD*	QTc duration	PLN*	ECV fraction*	H-FABP	
	QT dispersion	TTNtv*	Native T1 mapping	Galectin-3	
	Late potentials (SAECG)*	DES*	Papillary muscle scarring*	MMP-2, TIMP-1, and OPN *	
	HRV	TMEM43*	GLS*	Metabolomics	
	Heart rate turbulence	RBM20*	LV torsion *		
	Deceleration capacity	Polygenic risk scores	RV/LV blood pool (T2 mapping) *		

Overview of SCD risk stratification parameters in DCM. Asterisks (*) indicate markers associated with additional, increased risk of ventricular arrhythmias. DCM, Dilated cardiomyopathy; SCD, sudden cardiac death; CMR, cardiac magnetic resonance; ECG, electrocardiographic; NIRFs, Holter-derived non-invasive risk factors; NYHA, New York Heart Association; LVEF, Left Ventricular Ejection Fraction; PVC, Premature Ventricular Contraction; NSVT, Non-sustained Ventricular Tachycardia; TWA, T-wave alternans; SAECG, Signal-Averaged Electrocardiography; HRV, Heart Rate Variability; LMNA, lamin A/C; FLNC, filamin C; DSP, desmoplakin; TTNtv, titin truncating variants; DES, desmin; TMEM43, transmembrane protein 43; RBM20, RNA binding motif protein 20; *PLN*, Phospholamban; LGE, Late Gadolinium Enhancement; ECV, Extracellular Volume; GLS, Global Longitudinal Strain; RV, Right Ventricle; LV, Left Ventricle; NT-proBNP, N-terminal pro-B-type Natriuretic Peptide; sST2, soluble Suppression of Tumorigenesis-2; H-FABP, Heart-type Fatty Acid Binding Protein; MMP-2, Matrix Metalloproteinase-2; TIMP-1, Tissue Inhibitor of Metalloproteinase-1; OPN, Osteopontin; VT, Ventricular Tachycardia; VF, Ventricular Fibrillation; EPS, electrophysiological study.

### 3.2 The Role of ECG/Non-Invasive Risk Factors

Electrocardiographic (ECG) and Holter-derived non-invasive risk factors (NIRFs), including ventricular extrasystoles, late potentials, QT interval abnormalities, T-wave alternans (TWA), and heart rate variability (HRV) measures, offer widely accessible tools for assessing arrhythmic risk in patients with DCM, although their predictive value varies considerably and remains limited when used in isolation [25].

PVCs and NSVT are commonly detected on 24-hour ambulatory ECG monitoring, with reported prevalences of approximately 80–95% and 43–66%, respectively [26]. Given their high frequency in DCM, their utility as markers of arrhythmogenic substrate and predictors of SCD has been extensively investigated. Evidence supporting PVC burden as a prognostic marker is inconsistent. In a large cohort of 742 DCM patients with LVEF <35%, Setti et al. [27] reported a 1.31-fold increase of SCD or malignant VAs in patients with NSVT compared to those without, but did not show a statistically significant correlation between PVCs and SCD or major ventricular arrhythmias (MVAs). Data from a subgroup analysis of the DANISH trial concluded that NSVT and high-burdenPVCs (≥30/hour) were independently associated with increased all-cause and cardiovascular mortality, but not with SCD. In addition, neither marker conferred a survival benefit from ICD therapy [28]. Genotype-specific analyses suggest that PVC burden may have greater relevance in genetically mediated disease; one study reported that both NSVT and >500 PVCs/24 h were associated with MVAs in patients carrying pathogenic or likely pathogenic variants, while in non-genetic DCM, only NSVT retained prognostic significance [29]. Collectively, these findings indicate that while NSVT may reflect increased arrhythmic vulnerability, the high prevalence of both NSVT and PVCs in DCM limits their discriminative power.

Signal-averaged ECG (SAECG) enables the detection of ventricular late potentials (LPs), reflecting areas of slow conduction and potential arrhythmogenic substrate [30]. Although its use has declined in contemporary practice, several early studies reported higher rates of SCD, cardiac mortality, and all-cause mortality in DCM patients with abnormal SAECG findings [31,32,33]. In contrast, other investigations failed to confirm a significant association between late potentials and arrhythmic outcomes, due to low sensitivity and limited positive predictive value [34,35].

QT interval abnormalities, including QTc prolongation and increased QT dispersion, reflect repolarization heterogeneity and have been associated with malignant VAs in other cardiac conditions. At the same time, results in DCM populations remain conflicting and current evidence may not be enough to support routine use of these indices for SCD risk stratification. Studies using 12-lead ECG generally found no association between QTc duration and ventricular arrhythmias, cardiac death, or all-cause mortality [36,37]. Conversely, QTc measured during Holter monitoring was associated with increased VT/VF risk in broader heart failure populations, suggesting that dynamic assessment may be more precise [38]. In pediatric DCM, marked QTc prolongation (>510 ms) was associated with a significantly increased risk of life-threatening arrhythmias [39]. Similarly, studies assessing QT dispersion yielded inconsistent results, with some reporting increased SCD risk at higher values and others finding no prognostic value [36,40,41].

TWA, a marker of beat-to-beat repolarization instability, has shown more consistent prognostic performance. In the largest study to date, including 446 patients with NICM, abnormal TWA testing was associated with a fourfold increased risk of cardiac death or life-threatening arrhythmias and demonstrated a high negative predictive value [42]. While some studies failed to replicate these findings, a meta-analysis concluded that abnormal TWA is associated with a 3.25-fold increased risk of SCD in NICM [43,44]. Importantly, TWA appears particularly useful for identifying low-risk patients.

Autonomic markers have also been explored. Reduced HRV has been associated with increased cardiac mortality in several studies, including a substudy of the DEFINITE trial, although other investigations and meta-analyses failed to demonstrate predictive value for arrhythmic events [43,44,45,46]. Another marker reflecting the autonomic nervous system is heart rate turbulence, but it has not shown prognostic utility in DCM [47,48]. Finally, deceleration capacity has emerged as a potentially promising marker, with lower values associated with increased mortality, although based on limited data [49,50].

Overall, even though ECG- and Holter-derived NIRFs provide valuable insights into the electrophysiological and autonomic substrate of DCM, the available evidence is heterogeneous, largely derived from older studies, and demonstrates modest predictive performance when these markers are applied individually.

### 3.3 The Role of Genetics

Genetic testing is increasingly used in SCD risk stratification in DCM patients to identify these patients who might benefit more from an early prophylactic approach. A genetic cause is identified in up to 40% of DCM cases and even more commonly, in familial disease, with truncating *TTN* variants (*TTNtv*) being the most frequent mutations in up to 25% of cases [51,52]. The latest ESC Guidelines on the management of cardiomyopathies identify several highly arrhythmogenic genetic variants in genes encoding cytoskeletal, sarcomeric, and desmosomal proteins, including *FLNC*, *DES*, *DSP*, *PLN*, *LMNA*, *TMEM43*, and *RBM20* [23]. Results from a network meta-analysis of 26 studies suggest that the presence of pathogenic or likely pathogenic *TTNtv*, *LMNA* and desmosomal mutations was linked to a higher risk for composite adverse events, including all-cause mortality, heart failure, SCD, sustained VT and VF; however, only the *LMNA* and desmosomal groups were at increased risk of MVAs [53]. Indeed, lamin A/C (*LMNA*)-related cardiomyopathy remains the most well-established example where genotype directly guides clinical decision-making. *LMNA* mutation carriers exhibit a high incidence of SCD driven by VAs and advanced conduction disease, prompting guideline recommendations for early ICD consideration in the presence of high-risk features, even when LVEF is mildly or moderately affected [6,54]. Similarly, truncating filamin-C variants (*FLNCtv*) have been associated with a high incidence of life-threatening arrhythmias and SCD, irrespective of LVEF, warranting a stricter risk stratification approach [55].

### 3.4 The Role of Cardiac Magnetic Resonance

Advanced cardiac imaging with CMR has reshaped SCD risk stratification in NICM patients. The presence of LGE is widely accepted as a key predictor of major VAs and SCD —even in the presence of relatively preserved LVEF— with specific factors including LGE percent, distribution and patterns [56,57,58,59]. In a study of 1105 patients by Zhou et al. [60], LGE ≥7.2% was associated with increased SCD risk with an estimated HR of 4.75 (95% confidence interval: 2.91, 7.74; *p* < 0.001). These results were validated by another cohort of 1272 patients with DCM and LVEF <35%, with LGE ≥7.5% being associated with a 4.1-fold increase in SCD. Interestingly, the integration of LGE to clinical factors and most importantly LVEF, may hold great promise against SCD risk prediction in this population [61].

LGE distribution also carries a prognostic role in SCD risk stratification. Specific patterns, including subepicardial, ring-like, septal, or multiple-site involvement, have been linked to higher arrhythmic risk [62,63,64]. Importantly, even in patients with mildly or moderately reduced LVEF, the presence of midwall LGE identifies a high-risk subgroup for SCD [65]. A recent study by de Frutos et al. [66] demonstrated significant variations in LGE distribution across different genetic backgrounds and showed that linear midwall, subepicardial, transmural, and RV insertion LGE patterns were strongly associated with increased risks of major VAs and mortality.

Several non-LGE features have also been associated with an increased risk of adverse events and SCD. Native T1 mapping and extracellular volume (ECV) are elevated in DCM patients, reflecting diffuse interstitial fibrosis even when LGE is absent, making them potentially valuable early prognostic markers of the disease [67]. Zhou et al*.* [60] demonstrated that an ECV fraction ≥31.8% was linked to a 2.9-fold higher risk of SCD-related events (95% confidence interval (CI) 1.63–5.22; *p* = 0.001), while Rubiś et al. [68] reported that a cut-off value of 31.05% in global ECV was associated with increased VAs. Papillary muscle abnormalities, identified by signal hypointensity on cine images or scarring on dark-blood sequences, have also been associated with higher event rates [69,70]. CMR-derived global longitudinal strain (GLS) may also carry prognostic value, with independent meta-analyses suggesting significant associations with SCD in NICM patients, reporting hazard ratios of 1.27 (95% CI 1.10–1.46; *p* = 0.001) and 1.15 (95% CI 1.07–1.23; *p* < 0.001), respectively [71,72]. Less extensively studied CMR markers include right-to-left ventricular (RV/LV) blood pool on T2 mapping and left ventricular torsion [73,74]. Despite these promising findings, most non-LGE CMR markers should currently be considered investigational, as their incremental value beyond LGE and clinical parameters has not yet been consistently validated in large prospective cohorts.

Recent risk stratification models incorporating CMR parameters have been developed, such as the one by Zhou et al. [75]. The researchers combined NYHA class, LVEF, ECV, and LGE and demonstrated that patients with a mean ECV fraction ≥32.8% and LGE ≥5.9% were at increased risk of SCD, with LGE remaining the strongest predictor.

### 3.5 The Role of Biomarkers

Several traditional and emerging biomarkers have been investigated as potential tools to improve risk prediction in patients with DCM. NT-proBNP remains a fundamental biomarker for evaluating HF-related outcomes across all HF phenotypes, including DCM, with multiple studies showing that elevated NT-proBNP levels are associated with increased morbidity and mortality, particularly when LV dysfunction coexists [76,77,78]. Recent evidence has also highlighted the prognostic value of soluble ST2 (sST2), a decoy receptor of ST2, a molecule within the interleukin-1 receptor family. In a Polish cohort of patients with NICM, each unit increase in log-transformed ST2 was associated with a 2.7-fold higher risk of death (95% CI 1.32–5.53; *p* = 0.006), underscoring its potential utility in refining risk prediction [79].

Markers of myocardial injury, including high-sensitivity troponin T (hs-TnT), have consistently been shown to be elevated in DCM patients who experience adverse outcomes such as arrhythmic events and SCD [80]. Another myocyte injury marker, Heart-type fatty acid-binding protein (H-FABP), showed positive correlation with critical cardiac events, even though SCD was not observed in the sample group, rendering this specific marker a good indicator of overall cardiac risk in DCM patients and paving the way for future studies [81].

Inflammatory and fibrosis-related biomarkers have also gained attention. Galectin-3, a beta-galactosidase-binding lectin, is an inflammatory marker associated with myocardial fibrosis. Its serum levels have been closely correlated with the percent of LGE on CMR in patients with NICM, supporting its role in identifying individuals with higher fibrotic burden and therefore, higher arrhythmic risk [82]. Similarly, extracellular matrix turnover markers—including Matrix Metalloproteinase-2 (MMP-2), Tissue Inhibitor of Metalloproteinase-1 (TIMP-1), Growth Differentiation Factor 15 (GDF-15), and osteopontin (OPN)—have been associated with adverse events in DCM, including ventricular arrhythmias and SCD [83,84,85]. Among these, GDF-15 is the most prominent one, with many studies confirming its role in the prediction of arrhythmic events [84,85]. Another matrix enzyme, MMP-3, was linked to an increased incidence of major cardiac events, including SCD in DCM patients, particularly when combined with increased BNP levels [86].

Beyond traditional biomarkers, metabolomics offers a distinguished approach for identifying novel biomarkers in DCM, aiding in disease diagnosis, classification and prognosis. These metabolites are commonly associated with disrupted amino acid, energy and lipid pathways, though current evidence remains limited by small, case-control studies requiring larger multicenter validation [87]. One such study revealed that nuclear magnetic resonance spectroscopy-based metabolomic profiling, including metabolites such as creatine, creatinine, lactate and lipoprotein-related parameters, not only discriminates DCM survivors from non-survivors with 71% accuracy, but may also outperform NT-proBNP in predicting mortality risk, particularly when integrated with LVEF [88]. However, metabolomics remains an emerging field, and its clinical application in SCD risk stratification is currently limited by small study populations, lack of standardization, and the need for large prospective validation studies.

### 3.6 The Role of Programmed Ventricular Stimulation

Programmed ventricular stimulation (PVS) has been investigated as an adjunctive tool for SCD risk stratification in patients with DCM, particularly in those without clear indications for ICD therapy [89]. Earlier studies indicated a limited role of positive PVS in SCD risk stratification, demonstrating that inducible VAs alone were insufficient to reliably predict appropriate ICD therapy [90,91]. However, subsequent studies have reported contrasting findings, supporting the incremental value of PVS in selected patient populations. A study by Gatzoulis et al. [92] evaluated 158 patients with idiopathic DCM who underwent PVS using a standardized protocol and were offered an ICD in the presence of inducible VT/VF, recurrent syncope, or an indication for biventricular pacing. Among the 69 patients who received an ICD, the incidence of first appropriate ICD activation was approximately fourfold higher in the inducible compared with the non-inducible group [92]. Another study, which included 42 DCM patients with LVEF ≥40%, syncope, presyncope or positive NIRFs, concluded that PVS demonstrated a consistently high negative predictive value (100%) across all subgroups, while positive predictive value was highest in asymptomatic patients and in those with induced VF [93]. These results suggest that arrhythmia inducibility may identify a subset of DCM patients at markedly increased risk of appropriate ICD therapy, whereas the absence of inducible arrhythmias is associated with a lower arrhythmic risk [93].

In the era of advanced non-invasive testing and CMR-based risk stratification, the role of invasive electrophysiological study (EPS) has been substantially reduced in current ESC guidelines for the management of cardiomyopathies, in contrast to the 2022 guidelines for ventricular arrhythmias and SCD [6,23]. Nonetheless, EPS may have a complementary role in specific clinical scenarios, particularly when a two-step, multifactorial approach is pursued. In a study by Mueller et al. [94], inducibility of VT/VF during PVS was significantly associated with the presence of LGE on CMR in NICM patients. Additionally, in patients with structural heart disease (SHD) and unexplained syncope, EPS remains the only modality capable of assessing the integrity of the cardiac conduction system and identifying sinus node and/or atrioventricular node dysfunction, as well as malignant VAs, as potential mechanisms of loss of consciousness [95,96]. In such contexts, PVS may hold value when integrated with clinical features, imaging findings, and genetic data within a multiparametric risk stratification approach [97].

## 4. The Integrative Approach

Taking into consideration the heterogeneity in DCM phenotypes and the complex substrate behind VAs, several researchers have shifted towards an integrative approach for SCD risk stratification, involving clinical, electrocardiographic, imaging and genetic factors. Towards this direction, current ESC guidelines proposed several disease-specific arrhythmic risk scores for types of cardiomyopathies in the DCM spectrum, including the LMNA-risk VTA calculator, PLN risk calculator, and DSP risk score, which integrate easily accessible clinical factors with ECG and imaging parameters [23,98]. In addition, the DCM-SVA risk calculator was developed using seven parameters (sex, history of NSVT, history of syncope, family history of cardiomyopathy, QRS duration, LVEF and history of atrial fibrillation) and was able to predict the 5-year sustained VAs rate with increased accuracy, and reduce ICD implantations by 15%, compared to traditional guidelines. Adding LGE as an eighth parameter resulted in an increased risk of sustained VAs by about 72% beyond the original clinical model [99]. The integration of imaging and genetics seems to be a particularly favored approach, as specific LGE patterns might be correlated with specific gene mutations and guide disease phenotyping, prognosis and targeted treatment [100]. One such model was proposed by Mirelis et al. [101], who suggested that during a follow-up of almost 3 years, patients with either LGE or a pathogenic variant had significantly higher risks of malignant VAs and end-stage heart failure, with risks rising progressively from LGE (–)/genotype(–) to LGE (+)/genotype(+) patients (HR = 4.71, 95% CI: 2.11–10.50, *p* < 0.001 for VAs and HR = 7.92, 95% CI: 1.86–33.78, *p* < 0.001). Nevertheless, with the exception of the Arrhythmogenic Right Ventricular Cardiomyopathy (ARVC) risk calculator, PVS remains absent from existing cardiomyopathy risk models, despite its potential prognostic relevance, as discussed previously [102].

## 5. Recent Advances

Risk stratification in DCM is rapidly evolving through the integration of clinical, genetic, imaging, and computational tools, aiming to improve the identification of patients at high risk for adverse outcomes beyond the traditional LVEF-based approach. In the field of genetics, focus is shifting from gene-specific risk scores to variant-specific risk scores and recommendations [103,104]. In addition, research has shown that moving beyond the search for a monogenic disease etiology might lead to better characterization of arrhythmic risk in cardiomyopathies and improved patient outcomes. For this purpose, polygenic risk scores (PRS), that integrate numerous common genetic variants identified through genome-wide sequencing into a single quantitative measure of disease susceptibility, may be useful [105]. Moreover, the development of virtual EPS may assist in SCD risk stratification approaches and replace traditional invasive EPS. Virtual EPS encompasses computational methods and CMR or CT imaging to generate accurate whole-heart simulations, identify fibrotic areas, investigate mechanisms of arrhythmogenesis and assess arrhythmic vulnerability for sudden death risk prediction [106,107].

In parallel with advances in computational modelling such as virtual EPS, lately artificial intelligence (AI) and machine learning approaches are increasingly being applied to DCM patients, aiming to deliver personalized risk prediction models, particularly for patients with no clear ICD indications. Shu et al. [108] developed a machine learning model based on clinical and CMR features of DCM patients, to accurately predict all-cause mortality and heart transplantation. In another more focused approach, the Deep ARrhythmic Prevention in dilated cardiomyopathy (DARP-D) model and the DEEP RISK model combined LGE, ECG and clinical data and predicted arrhythmic risk in NICM with improved accuracy [109,110]. In a similar approach, other researchers have developed a deep neural network (DNN) model to predict MVAs in DCM patients from ECG-derived parameters, which highlighted a potential value of P-wave related indices as a marker of abnormal electrophysiological substrate in this patients [111]. Nevertheless, these approaches remain largely investigational and require prospective validation before routine clinical implementation.

## 6. Ongoing Research

Building on these advances, several ongoing studies are aiming to further refine risk stratification strategies in DCM. Various RCTs are currently being conducted, with the ultimate goal of redefining the criteria for prophylactic ICD therapy and exploring novel biomarkers and imaging modalities. CMR-based approaches are gaining significant ground in the field of risk stratification. The CMR GUIDE trial (Clinicaltrials.gov identifier: NCT01918215) will assess fibrosis in relatively preserved LVEF patients (36–50%) and randomize them either to ICD therapy of implantable loop recorder [112]. The BRITISH trial (Clinicaltrials.gov identifier: NCT05568069) on the other hand, will focus on NICM patients with an LVEF ≤35% and based on the existence of scarring, patients will be randomized to an ICD approach or to an implantable loop recorder (ILR)/cardiac resynchronization therapy (CRT-P) approach [113]. Other studies are exploring integrative, electrophysiology-focused approaches that combine invasive and non-invasive data to enhance risk stratification and guide personalized management. In this field, the ReCONSIDER (A***r***rhythmi***c*** risk stratificati***on*** in noni***s***chem***i***c ***d***ilat***e***d ca***r***diomyopathy) study is a multicenter, prospective, observational trial (Clinicaltrials.gov identifier: NCT04246450), which aims to test a two-step, multifactorial approach in patients with both mildly reduced (35% < LVEF ≤50%) and severely reduced (LVEF ≤35%) systolic function [114]. The protocol begins with assessment of NIRFs, including clinical, echocardiographic, ECG, Holter, and CMR markers, followed by invasive programmed ventricular stimulation, with inducible VT or VF defining a positive study. Patients with LVEF ≤35% and LVEF between 35–50% with inducible VT/VF plus 1 NIRF will receive ICD therapy. ReCONSIDER aims to compare CMR–based and electrophysiology-driven strategies capable of serving as the initial step in a two-step approach, in order to improve SCD risk stratification and survival.

## 7. Challenges and Future Considerations

Despite significant advances in risk stratification, several important challenges and unresolved issues in the management of patients with DCM should be acknowledged. First, it remains uncertain how contemporary guideline-directed medical therapy (GDMT) will alter the natural history of the disease, particularly in the context of significant improvements in LVEF observed with novel pharmacological agents and the potential consequent reduction in arrhythmic risk [115]. Second, the absence of a clear benefit of ICD therapy in the early disease phase should not be interpreted as a lack of long-term benefit, as relatively few studies have evaluated extended follow-up outcomes. Notably, long-term follow-up of the DANISH trial, with a median duration of up to 13.2 years, demonstrated a 46% reduction in SCD among ICD recipients, particularly in patients aged ≤70 years [116]. Third, previous evidence has shown that arrhythmic risk is dynamic and may be influenced by disease progression, reverse remodeling and other comorbidities, underscoring the need for close monitoring and repeated risk assessment of these patients [117].

Genetic testing also introduces important clinical dilemmas, including the interpretation of variants of uncertain significance (VUS) and the identification of pathogenic variants in patients with mild phenotypes, where the cost–benefit of prophylactic ICD therapy should be carefully balanced [118,119]. This challenge has been partially addressed by the incorporation of genetic testing into clinical, ECG, Holter monitoring or imaging features to create a genotype-phenotype model that may allow for a more personalized risk assessment and improved identification of individuals at increased risk of MVAs or SCD [27,120]. However, although several multifactorial risk calculators have shown promising results, most have been derived from relatively small cohorts, with the notable exception of the *LMNA* risk calculator. Thus, careful external validation and standardization across diverse populations and socioeconomic settings are required to ensure equal application, clinical relevance and, ultimately, improved patient outcomes.

Beyond these points, accurate risk stratification is further challenged by the marked heterogeneity of the NICM population, as mentioned previously. The emerging entity of non-dilated left ventricular cardiomyopathy (NDLVC) substantially overlaps with traditional DCM and is characterized by absence of or only mild left ventricular dilatation and dysfunction. Nevertheless, these patients share similar clinical characteristics and remain at risk for adverse outcomes, while reliable predictors of SCD have yet to be established [121,122,123]. In addition, in patients with high PVC burden, it is essential to distinguish true NICM related to structural heart disease from PVC-induced cardiomyopathy, as prognosis differs substantially between these entities [124,125].

## 8. Clinical Implications and Device-Based Management

From a clinical perspective, the optimal selection of cardiac implantable electronic devices (CIEDs) remains a key challenge. In patients who meet guideline indications for cardiac resynchronization, device selection between CRT-D and CRT-P remains clinically relevant. Emerging data indicate that CMR tissue characterization, genotype status and adjunctive electrophysiologic testing can refine CRT-D decision-making beyond LVEF and QRS duration alone, helping identify patients who may truly benefit from defibrillator therapy while sparing low-risk individuals unnecessary ICD implantation [126].

In parallel, conduction system pacing, including His-bundle pacing and left bundle branch area pacing, has emerged as a more physiological alternative to traditional biventricular pacing in patients with electrical dyssynchrony and may have a role in selected heart failure populations [127,128,129].

Another consideration, particularly in younger patients or those without pacing needs, is the avoidance of transvenous ICD systems in favor of subcutaneous (S-ICD) or extravascular ICD (EV-ICD) technology. Although evidence for EV-ICDs is still emerging, recent data from the Greek Analysis of the Subcutaneous-ICD Practice (GASP) registry demonstrated high efficacy and a low acute complication rate with S-ICDs across a heterogeneous cohort, with the notable exception that none of the included DCM patients experienced inappropriate shocks [130,131].

In specific scenarios, such as newly diagnosed DCM or during optimization of guideline-directed medical therapy while awaiting potential LVEF recovery, temporary strategies may be considered. The placement of automated external defibrillators (AEDs) in the homes of patients at high risk of SCD, accompanied by training of family members or caregivers, has been proposed; however, the cost-effectiveness of this approach and its impact on long-term clinical outcomes remain uncertain [132].

Collectively, these therapeutic dilemmas further emphasize the need for individualized SCD risk stratification in DCM and reinforce the potential value of multiparametric models to guide device selection and improve patient outcomes.

## 9. Conclusion

The complex substrate and the heterogeneity of the DCM make accurate risk stratification for SCD a challenge. The transition from solely relying on LVEF to a multiparametric model, including advanced imaging and genetic features, marks a paradigm shift in personalized cardiomyopathy care. Emerging tools hold promise for further refining risk prediction and guiding therapeutic decisions, while future research, particularly large randomized trials, is essential to redefine the criteria for primary SCD prevention in this group of patients.
